# Risk relationship between osteoporosis and plasma proteins

**DOI:** 10.1097/MD.0000000000044105

**Published:** 2025-08-29

**Authors:** Cai Chen, Qin Zeng, Qianling Ye, Futai Jin

**Affiliations:** a Department of Education, Dongguan Hospital of Traditional Chinese Medicine, Dongguan, Guangdong Province, China; b Department of Orthopedics, Dongguan Hospital of Traditional Chinese Medicine, Dongguan, Guangdong Province, China; c College of Traditional Chinese Medicine, Guangdong Pharmaceutical University, Guangzhou, Guangdong Province, China; d Guangzhou University of Chinese Medicine Graduate School, Guangzhou, Guangdong Province, China.

**Keywords:** Mendelian randomization, OP, plasma proteins, therapeutic targets

## Abstract

Osteoporosis (OP) has gradually become a major public health problem. It is clinically important to elucidate further the pathophysiologic mechanisms that induce OP and to identify more effective therapeutic targets. In the present study, we used Mendelian randomization analysis to assess the causal effects of 4907 plasma proteins on bone mineral density (BMD) outcomes to identify their potential therapeutic targets. The data files of 4907 plasma proteins were downloaded from the deCODE Genetics database, the bone density data files were obtained from the publicly available genome-wide association study database, the single nucleotide polymorphisms of weak instrumental variables in plasma proteins were removed based on the *F* test value of >10, the inverse variance weighted method was the main statistical method, and MR-Egger analysis was used for the test of diversity and difference. The obtained plasma proteins strongly associated with the disease were enriched by gene ontology (GO), pathway enrichment, and protein interaction networks were constructed in the GeneMANIA database. Based on *P* < .05 and false discovery rate value < 0.2, plasma proteins with consistent odds ratio values of 5 statistical methods were extracted, and a total of 22 plasma proteins strongly associated with diseases were obtained. The results of GO enrichment analysis showed that the disease-associated plasma proteins were functionally related to the Notch signaling pathway, trabecular morphogenesis, and so on, and their main enrichment was in the Notch signaling pathway and Toll-like receptor signaling pathway. A total of 20 interacting genes were predicted by the GeneMANIA database. Through least absolute shrinkage and selection operator regression, key variables were selected from 22 plasma proteins. There is a causal relationship between plasma proteins and BMD, with proteins such as recombinant pleckstrin homology domain containing family A, member 1, RAB6B, member RAS oncogene family (RAB6B), and UDP-glucose dehydrogenase exacerbating the disease process. In contrast, proteins such as lipopolysaccharide-binding protein, manic fringe homolog, and cartilage adhesion protein can exert a protective effect, and both act as prognostic markers for OP and as potential therapeutic targets.

## 1. Introduction

Osteoporosis (OP) is an age-related bone disease that primarily affects postmenopausal women and is characterized by a systemic condition of decreased bone mineral density (BMD), altered bone microarchitecture, and increased bone fragility, which can lead to fractures.^[1,2]^ The prevalence of OP increases with age, and epidemiologic surveys show that more than 40 million women in the United States have been diagnosed with lowered bone density. As many as 400,000 hip fractures are caused by OP each year.^[3,4]^ With the aging of the population, there will be a sharp increase in the number of patients with OP in the future, which will bring a great economic burden to individuals, families, and society.^[5]^ Therefore, it is of great value to look for risk factors for its occurrence and to target prevention. As a systemic bone metabolic disease, OP often has no obvious clinical symptoms in the early stage and is usually detected only after a fracture occurs. It is imperative to predict OP promptly.^[6]^ For the treatment of OP, medication is currently used.^[7]^ The main drugs used in clinical practice to treat OP are bone formation enhancers, bone resorption inhibitors, and bone mineralizers. In contrast, bisphosphonates, estrogen receptor (selective) modulators, and calcitonin commonly alleviate the symptoms. Although effective, they all have some problems.^[8]^ For example, bisphosphonates are associated with adverse effects such as gastrointestinal reactions, arthralgia, myalgia, and osteonecrosis of the jaw; in addition, some bisphosphonates have been reported to cause a range of nonnegligible side effects, such as an increased risk of venous thromboembolism and esophageal cancer.^[8,9]^ Estrogen receptor (selective) modulators, despite their significant therapeutic effect in the early stages of OP, increase the risk of cardiovascular disease with long-term use.^[10]^ Calcitonin is therapeutic in OP by regulating bone metabolism and inhibiting osteoclast activity. Still, it can also cause adverse effects such as limb pain, circulation disorders, and severe stroke.^[11]^ Given the limitations of the side effects of many of the current drugs for OP, the search for new therapeutic targets for OP will not only help to address the limitations of the current treatments but also provide an essential direction for a deeper understanding of the disease mechanisms, precision medicine, and future drug development.

Blood is the most commonly used medium for clinical analysis, rich in a variety of proteins, and is currently an important biofluid for the study of biomarkers for the diagnosis and prognosis of human diseases.^[12]^ Drugs approved by the US Food and Drug Administration in 2017 all targeted human proteins, and plasma proteins play a central role in a range of biological processes (BPs), as well as in a variety of diseases, according to the results of a survey.^[13]^ Genome-wide association studies (GWAS) are a research strategy to study phenotype–genotype relationships at the population level. Currently, it has identified more than 100,000 single-nucleotide polymorphisms (SNPs) associated with human disease.^[14]^ Plasma proteins are well-studied traits in GWAS because they represent an intermediate layer between genetic variation and disease progression. Several studies have identified protein quantitative trait loci (pQTL) in population-based cohorts and linked their findings to downstream disease risk.^[15]^ Several recent studies have revealed a strong link between plasma proteins and OP. A study found that plasma fibroblast growth factor 23 levels before heart transplantation were positively correlated with BMD scores in the lumbar spine after transplantation.^[16]^ Another study found that intermittent parathyroid hormone treatment enhanced the activity of the insulin-like growth factor system by upregulating pregnancy-associated plasma protein-A expression, thereby promoting bone formation.^[17]^ It has also been proposed that the Annexin A2 protein may reduce BMD by promoting the migration of peripheral blood monocytes to bone-resorbing surfaces and increasing osteoclastogenesis and bone resorption.^[18]^ More comprehensive and systematic evidence is needed to understand their relationship because of the complexity of plasma proteins and the increasing number of plasma proteins associated with OP.

**Figure 1.**
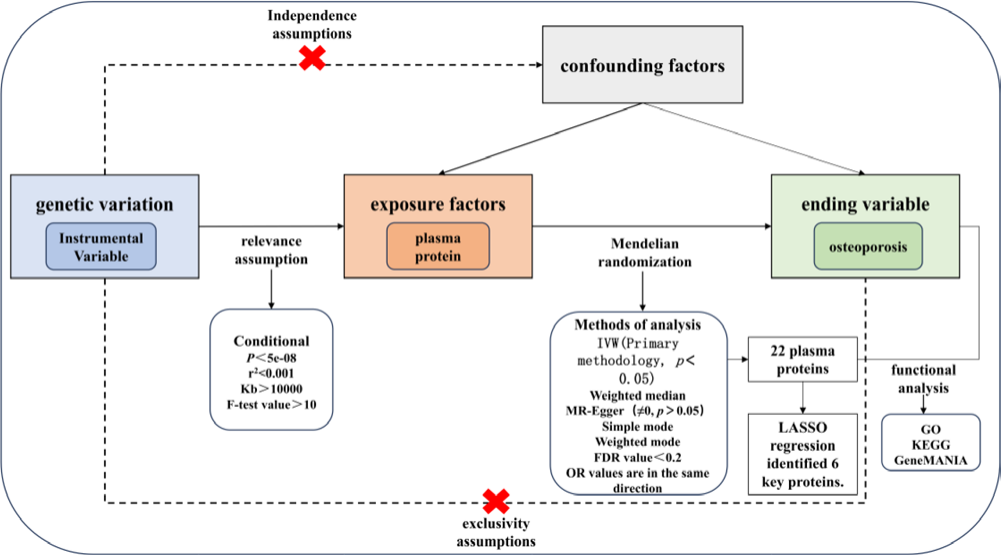
Article structure. FDR = false discovery rate, GO = gene ontology, IVW = inverse variance weighted, KEGG = pathway enrichment, LASSO = least absolute shrinkage and selection operator, MR = Mendelian randomization, OR = odds ratio.

Mendelian randomization (MR) is a novel analytical approach that uses genetic variation as an instrumental variable (IV) to assess the causal relationship between risk factors and outcomes.^[19]^ Compared with traditional observational studies, MR studies have the advantages of large sample sizes, less influence of unknown confounders, and reverse causality. Due to these advantages, MR has been widely used in research in recent years.^[20]^ In the present study, we analyzed the causal association between 4907 plasma proteins and BMD by MR study, and this study may help to reveal the genetic causality between 4907 plasma proteins and OP, which may help in the diagnosis and treatment of OP. The article structure of this paper is shown in Fig. 1.

## 2. Materials and methods

### 2.1. Exposure data sources

The 4907 plasma proteins used as exposure factors in this study stem from those identified by the deCODE Genetics research team in 2021, which utilized plasma proteomics to massively integrate genomic data between gene sequence variants and plasma protein expression levels in 35,559 Icelandic populations and found sequence variants in plasma pQTL and protein levels 18,084 associations between sequence variants in plasma pQTLs, in addition, the team tested plasma protein levels for associations with 373 diseases and other traits, identifying 257,490 associations.^[21]^ The human genetic data for the current study were obtained from the published GWAS in 2021, which had been applied for by the relevant ethical review board before the current research and, therefore, did not require additional ethical approval. This exposure file is from (https://www.decode.com/summarydata) database.

### 2.2. Sources of data on end results

Whole-body BMD was the outcome factor in this trial, which was derived from a study reported by Medina-Gomez et al^[22]^ in 2018, which contained 56,284 European population samples containing a total of 16,162,733 SNPs.

### 2.3. Removing weak IVs

IV selection and MR analyses were conducted through 3 hypotheses: IV are strongly associated with exposure factors; IV are independent of confounders; and IV only affect the outcome through exposure factors and are not directly associated with the outcome.^[23]^ SNPs strongly associated (*P* < 5e−8) were selected as IV, and chain imbalances were removed (set *r*^2^ = 0.001 and *kb* = 10000). The strength of IV was measured using the *F* test, calculated as *F* = *R*^2^ (*N* *−* *k* *−* 1)/*k*(1 − *R*^2^), where *R*^2^ represents the extent to which IV explains exposure, *N* represents the sample size of the GWAS study of exposure factors, and *k* represents the number of SNPs.^[24]^ Screening SNPs with *F* > 10 to quantify IV strength and indicate a strong correlation.^[25]^ We used the “TwoSampleMR” package and the “ieugwasr” package in the R software (Version 4.3.1) to filter the IVs that met the requirements.

### 2.4. MR analysis

All plasma protein exposure data files were read with the “VariantAnnotation” “gwasglue” “TwoSampleMR” package in R software, and information was extracted. The ending data ID was “ebi-a-GCST005348.” To assess the accuracy of the results, multiple sensitivity analyses were performed, including the MR-Egger method and the leave-one-out method. The MR-Egger method tests for horizontal multidirectionality and assesses whether IV affects outcomes through pathways other than exposure, and if *P* > .05 indicates no significant horizontal pleiotropy exists. The leave-one-out method evaluated the effect of individual SNPs on the overall MR estimate. Cochran *Q* test assessed heterogeneity, with *P* > .05 indicating no significant heterogeneity. The results were expressed as odds ratios (ORs) and 95% confidence intervals. Scatter plots, forest plots, and funnel plots were generated for key plasma proteins.

### 2.5. Screening for disease-associated plasma proteins

The results of the MR analysis and the “csv” file of the pleiotropy test were imported into the R language software, based on the inverse variance weighted (IVW) as the main statistical method, supplemented by the weighted median, MR-Egger, simple mode, weighted mode, and other 5 analytical techniques to screen out the same direction of OR value for the 5 analytical methods.

### 2.6. MR analysis of disease-related plasma proteins

To analyze the risk relationship between disease-related plasma proteins and BMD using MR, all the plasma protein exposure data files and BMD outcome data files were analyzed using the “TwoSampleMR” software package in R language software to obtain the risk relationship between disease-related plasma proteins and OP. A forest plot visualizing the relationship between plasma proteins and diseases was drawn using the “grid, readr, forestploter” software package.

### 2.7. Gene ontology and pathway enrichment analysis

We will use the “clusterProfiler,” “org. Hs. e.g.. db,” and “enrichplot” packages in the R language to perform gene ontology (GO) and pathway enrichment (KEGG) analysis on plasma proteins closely related to diseases under the screening conditions of *P* < .05 using the IVW method, and construct a visual bubble plot.

### 2.8. GeneMANIA database predicts plasma protein interaction genes

Using the database (http://genemania.org/), plasma proteins meeting the IVW test with a *P* value of <0.05 and the OR values of the 5 analytical methods in the same direction were predicted, and genes with interactions or regulatory roles were screened.

### 2.9. Least absolute shrinkage and selection operator regression screening feature variables

To further screen the related to disease proteins obtained from MR analysis, we used the least absolute shrinkage and selection operator (LASSO) regression to rank the importance of all feature variables and selected variables with higher importance. First, we downloaded the GSE56815 dataset from the NCBI database (https://www.ncbi.nlm.nih.gov/), which contains 40 control and 40 OP group samples. We took the average gene expression levels with the same symbol and obtained expression data of 17 corresponding genes out of the 22 plasma proteins from GSE56815. The data were homogenized within groups using the R software “base” package. Using the “glmnet” package in R software, we set the cross-validation coefficient to 10 and the regularization ratio to 1 to determine the optimal lambda value and screen key variables.

### 2.10. Statistical analysis

The results were expressed as OR and 95% confidence intervals, with *P* < .05 considered statistically significant. Differences between IVs existed if the Cochran *Q* statistic was *P* < .05, but not when *P* > .05. MR-Egger regression intercepts were considered horizontally multivariate if not 0 and *P* < .05. Conversely, they were considered not horizontally multivariate if *P* > .05.

## 3. Results

### 3.1. Removal of plasma protein files for weak IVs

Based on the original SNPs screening conditions (*P* < 5e−8, *r*^2^ = 0.001, *kb* = 10 000, *F* > 10), 31,315 SNPs closely related to the exposure factors were obtained using R language software.

### 3.2. Acquisition of plasma proteins strongly associated with disease

**Figure 2.**
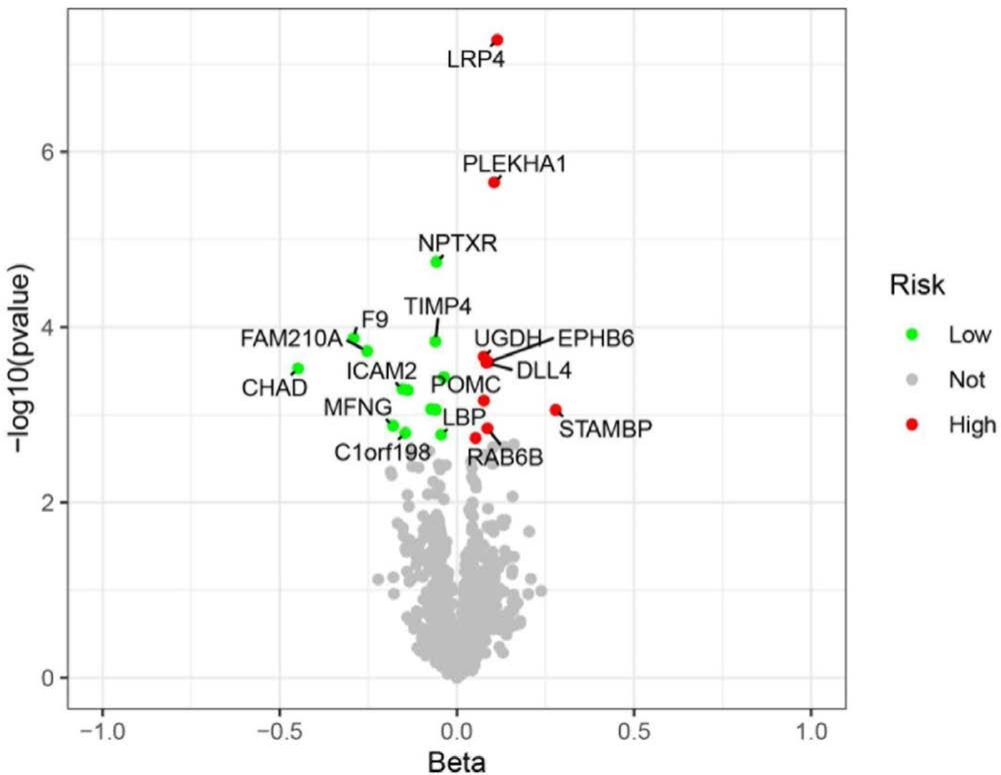
Volcanic map of plasma proteins strongly associated with 22 diseases. Horizontal coordinates represent the beta values of 22 plasma proteins, vertical coordinates are −log10(*P* value), and risk factors for BMD are labeled in red and protective factors for BMD in green. BMD = bone mineral density.

**Figure 3.**
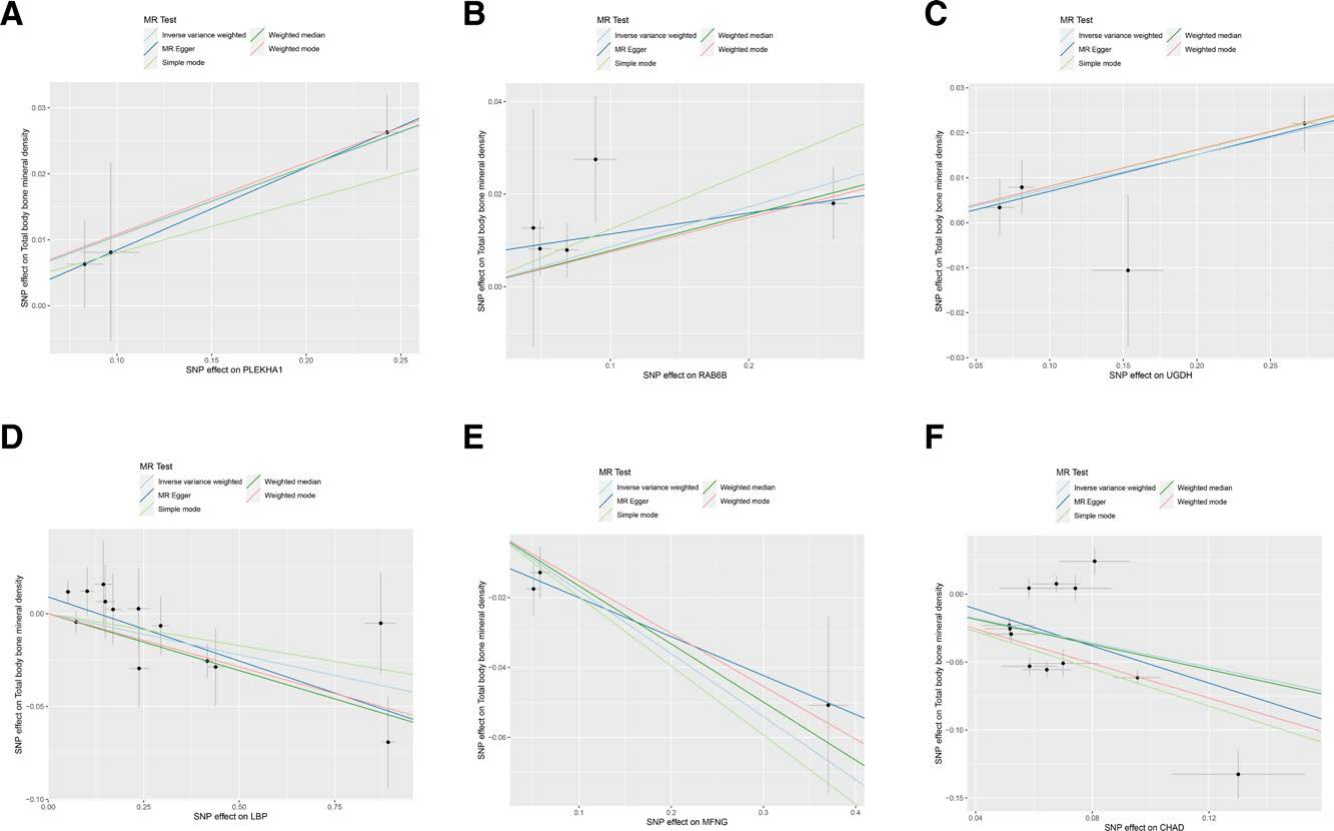
Scatterplot of causal associations between 6 key plasma proteins and osteoporosis. (A–F) represents PLEKHA1, RAB6B, UGDH, LBP, MFNG, CHAD, and osteoporosis. CHAD = cartilage adhesion protein, LBP = lipopolysaccharide-binding protein, MFNG = manic fringe homolog, MR = Mendelian randomization, PLEKHA1 = recombinant pleckstrin homology domain containing family A, RAB6B = RAB6B, member RAS oncogene family, SNP = single-nucleotide polymorphism, UGDH = UDP-glucose dehydrogenase.

**Figure 4.**
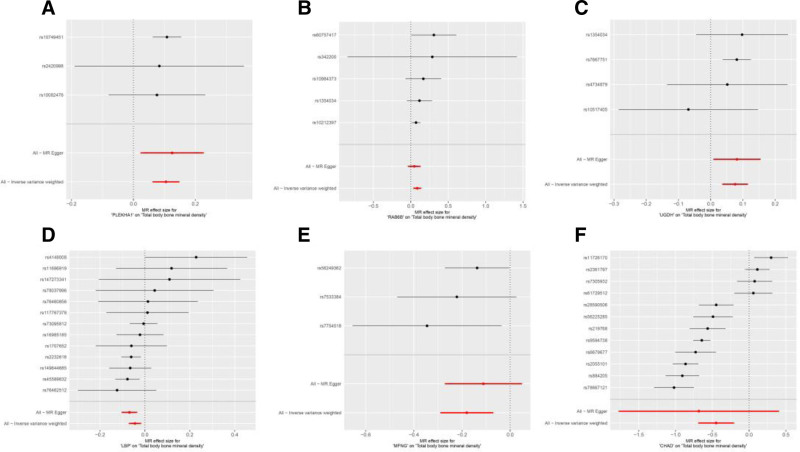
Forest plot of causal associations between 6 key plasma proteins and osteoporosis. (A–F) represents PLEKHA1, RAB6B, UGDH, LBP, MFNG, CHAD, and osteoporosis. CHAD = cartilage adhesion protein, LBP = lipopolysaccharide-binding protein, MFNG = manic fringe homolog, MR = Mendelian randomization, PLEKHA1 = recombinant pleckstrin homology domain containing family A, RAB6B = RAB6B, member RAS oncogene family, UGDH = UDP-glucose dehydrogenase.

**Figure 5.**
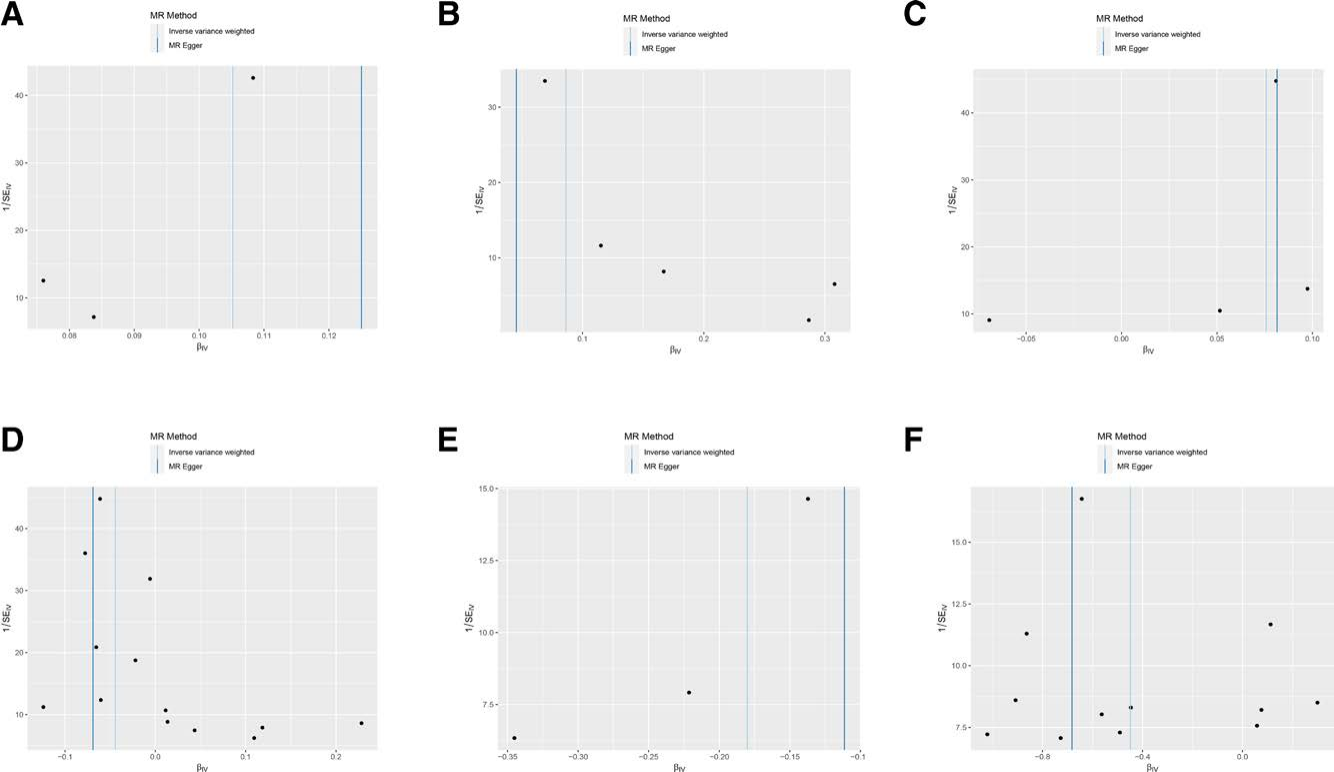
Funnel plot of causal associations between 6 key plasma proteins and osteoporosis. (A–F) represent PLEKHA1, RAB6B, UGDH, LBP, MFNG, CHAD, and osteoporosis. CHAD = cartilage adhesion protein, LBP = lipopolysaccharide-binding protein, MFNG = manic fringe homolog, MR = Mendelian randomization, PLEKHA1 = recombinant pleckstrin homology domain containing family A, RAB6B = RAB6B, member RAS oncogene family, UGDH = UDP-glucose dehydrogenase.

SNPs with *F* test value >10 and outcome data ID “ebi-a-GCST005348” were analyzed by MR analysis using R language software; the IVW method was the main statistical method, and the *P* value was set at <0.05. If the Cochran *Q* test suggested no heterogeneity, fixed effects IVW was chosen as the primary outcome; otherwise, random effects IVW was selected.^[26]^ A total of 227 plasma proteins related to diseases were obtained. Based on *P* < .05 and false discovery rate (FDR) value < 0.2, plasma proteins with consistent OR values of the 5 statistical methods were extracted, a total of 22 disease-related key plasma proteins were obtained, and the visualized volcano diagram was constructed. The red in the volcano diagram represents proteins at high risk of disease, and the green color represents proteins at low risk. We screened 5 representative proteins among the high- and low-risk proteins based on significance, respectively, for further discussion. Twenty-two plasma proteins were tested for heterogeneity and pleiotropy with OP; none 19 were heterogeneous or pleiotropy (*P* *>* .05). In contrast, Cochran *Q* statistic for LRP4, FAM210A, and cartilage adhesion protein (CHAD) suggested heterogeneity for the IVs (*P* < .05). Further assessment of the pleiotropy of IVs using MR-Egger resulted in no evidence of significant pleiotropy (*P* > .05), and no SNP was found to significantly affect the overall results in the leave-one-out sensitivity analysis, indicating reliable results. The results of the MR analysis are detailed in Table 1, and the volcano map is shown in Fig. 2. Detailed results of the tests for pleiotropy and heterogeneity are shown in Table 2. Scatterplots, forest plots, and funnel plots are shown in Figs. 3 and 4 with Fig. 5.

**Table 1:** Results of MR analysis of plasma proteins strongly associated with 22 diseases.

Plasma protein	Methodology	*β* Value	OR value	95% CI	FDR value
LRP4	IVW	0.114	1.121	1.076–1.168	0.000
PLEKHA1	IVW	0.105	1.111	1.064–1.160	0.003
NPTXR	IVW	−0.058	0.944	0.919–0.969	0.009
F9	IVW	−0.291	0.747	0.643–0.868	0.048
TIMP4	IVW	−0.061	0.941	0.912–0.971	0.048
FAM210A	IVW	−0.253	0.776	0.680–0.887	0.056
UGDH	IVW	0.076	1.079	1.036–1.123	0.057
EPHB6	IVW	0.089	1.093	1.042–1.146	0.057
DLL4	IVW	0.083	1.087	1.039–1.136	0.057
CHAD	IVW	−0.448	0.639	0.501–0.814	0.061
ST3GAL6	IVW	−0.036	0.964	0.945–0.984	0.066
ICAM2	IVW	−0.153	0.858	0.787–0.935	0.078
CXCL10	IVW	−0.138	0.871	0.805–0.942	0.078
POMC	IVW	0.076	1.079	1.033–1.128	0.096
BPIFA2	IVW	−0.073	0.930	0.891–0.971	0.108
ACP2	IVW	−0.060	0.942	0.910–0.976	0.108
STAMBP	IVW	0.279	1.322	1.121–1.558	0.108
MFNG	IVW	−0.180	0.835	0.748–0.932	0.148
RAB6B	IVW	0.086	1.090	1.034–1.149	0.154
C1orf198	IVW	−0.146	0.864	0.789–0.946	0.169
LBP	IVW	−0.044	0.957	0.931–0.983	0.171
ADAMTSL1	IVW	0.053	1.054	1.020–1.090	0.182

CI = confidence interval, FDR = false discovery rate, IVW = inverse variance weighted, MR = Mendelian randomization, OR = odds ratio.

**Table 2:** Heterogeneity and pleiotropy tests for MR analysis of 22 plasma proteins and OP.

Exposure factor	Outcome	Heterogeneity test				Pleiotropy test	
		IVW (Cochran *Q* test)	*P* value	MR-Egger (Cochran *Q* test)	*P* value	MR-Egger intercept	MR-Egger intercept *P* value
LRP4	OP	30.629	.022	27.592	.035	−0.007	.203
PLEKHA1	OP	0.176	.916	0.000	.996	−0.004	.747
NPTXR	OP	6.259	.618	6.257	.510	0.000	.974
F9	OP	4.297	.117	2.321	.128	0.018	.526
TIMP4	OP	7.676	.742	7.595	.668	0.001	.782
FAM210A	OP	10.095	.039	9.969	.019	−0.004	.858
UGDH	OP	1.934	.586	1.900	.387	−0.001	.871
EPHB6	OP	4.357	.628	3.508	.622	0.005	.399
DLL4	OP	3.102	.541	1.191	.755	−0.014	.261
CHAD	OP	186.579	.000	183.157	.000	0.016	.675
ST3GAL6	OP	23.573	.704	22.070	.734	−0.003	.231
ICAM2	OP	5.175	.270	5.017	.171	0.005	.779
CXCL10	OP	3.858	.426	3.299	.348	0.010	.528
POMC	OP	14.403	.420	14.207	.359	0.002	.679
BPIFA2	OP	13.795	.130	11.684	.166	0.006	.264
ACP2	OP	8.460	.294	7.973	.240	0.005	.567
STAMBP	OP	3.851	.278	0.665	.717	0.017	.216
MFNG	OP	1.597	.450	0.258	.612	−0.009	.454
RAB6B	OP	3.094	.542	1.642	.650	0.007	.315
C1orf198	OP	1.128	.569	0.069	.792	0.007	.491
LBP	OP	13.875	.309	9.691	.558	0.009	.065
ADAMTSL1	OP	8.881	.448	8.199	.414	−0.005	.438

IVW = inverse variance weighted, MR = Mendelian randomization, OP = osteoporosis.

### 3.3. MR analysis of 22 strongly correlated plasma proteins

**Figure 6.**
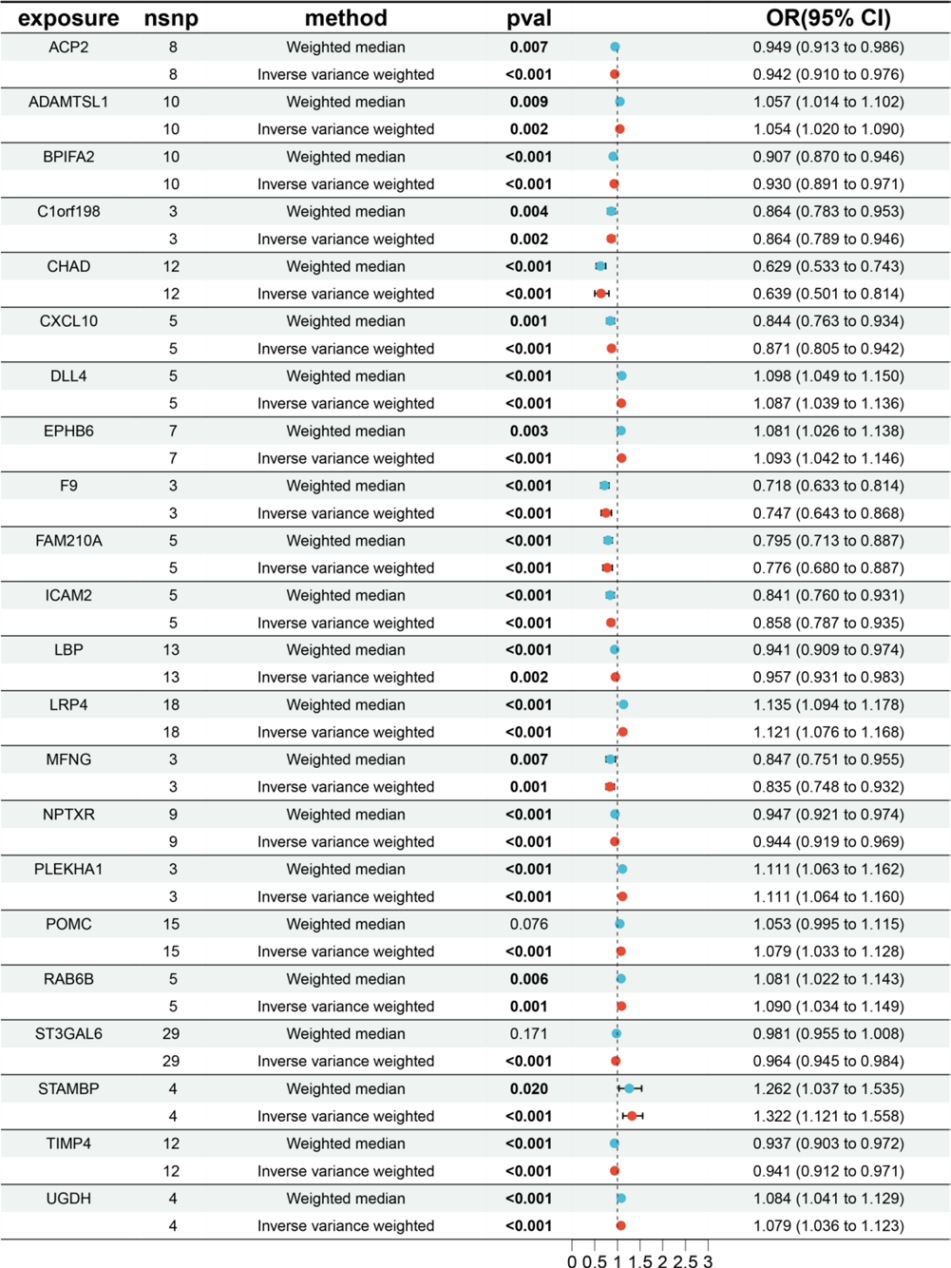
Forest map of plasma proteins strongly associated with 22 diseases. CI = confidence interval, OR = odds ratio.

MR analyzed the plasma proteins of 22 diseases based on the IVW method with *P* < 0.05, and all the “csv” files were obtained, and visual forest plots were constructed by using the installation packages of “grid, readr, forestploter” and so on in the R software. The forest plot is shown in Fig. 6.

### 3.4. GO and KEGG enrichment analysis

**Figure 7.**
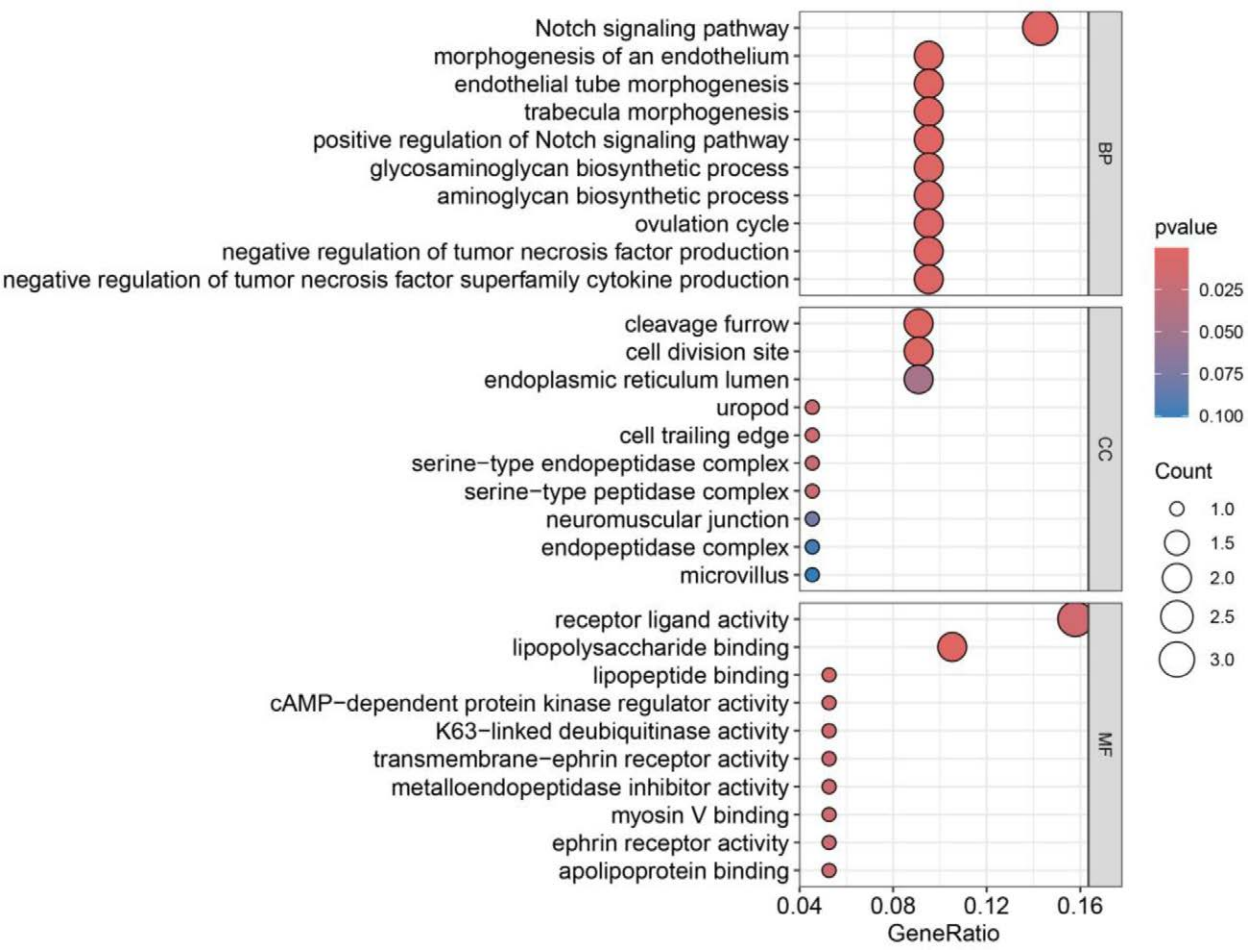
Bubble plots for GO enrichment analysis of plasma proteins strongly associated with 22 diseases. Darker colors represent more significant enrichment. GO = gene ontology.

**Figure 8.**
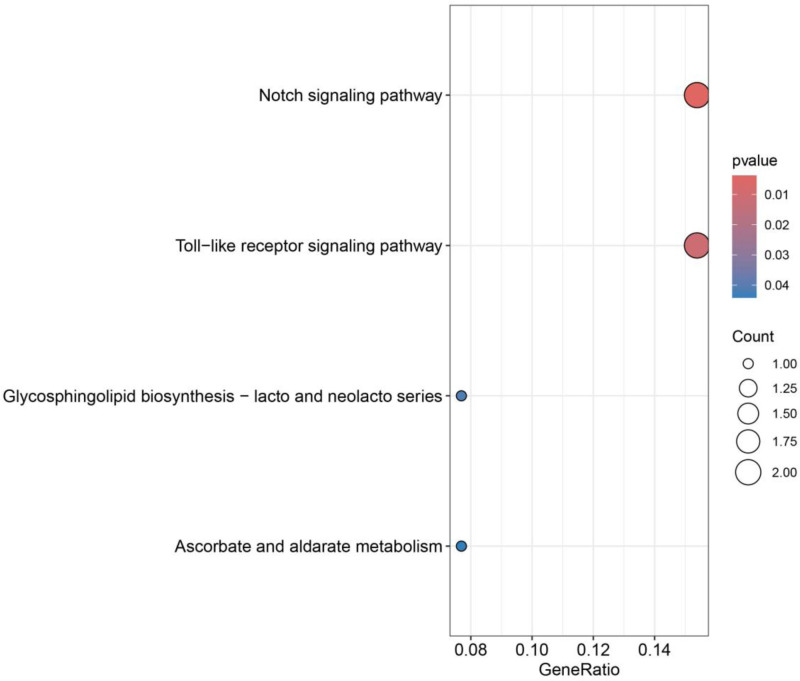
Bubble map of KEGG enrichment analysis of plasma proteins strongly associated with 22 diseases. Darker colors represent more significant enrichment. KEGG = pathway enrichment.

Twenty-two disease-associated plasma protein genes were subjected to KEGG and GO enrichment analysis, and a total of 167 BP, 7 cellular components, and 21 molecular function results were obtained based on a *P* < .05 as the threshold. The results of GO enrichment analysis showed that the biological functions of the genes were mainly involved in the Notch signaling pathway, trabecular morphogenesis, and other functions, and the results of KEGG enrichment analysis showed that the genes were primarily enriched in the Notch signaling, Toll-like receptors (TLRs) signaling, and so forth. The GO bubble diagram is shown in Fig. 7, and the KEGG bubble diagram is shown in Fig. 8.

### 3.5. Construction of an interaction network of 22 plasma proteins

**Figure 9.**
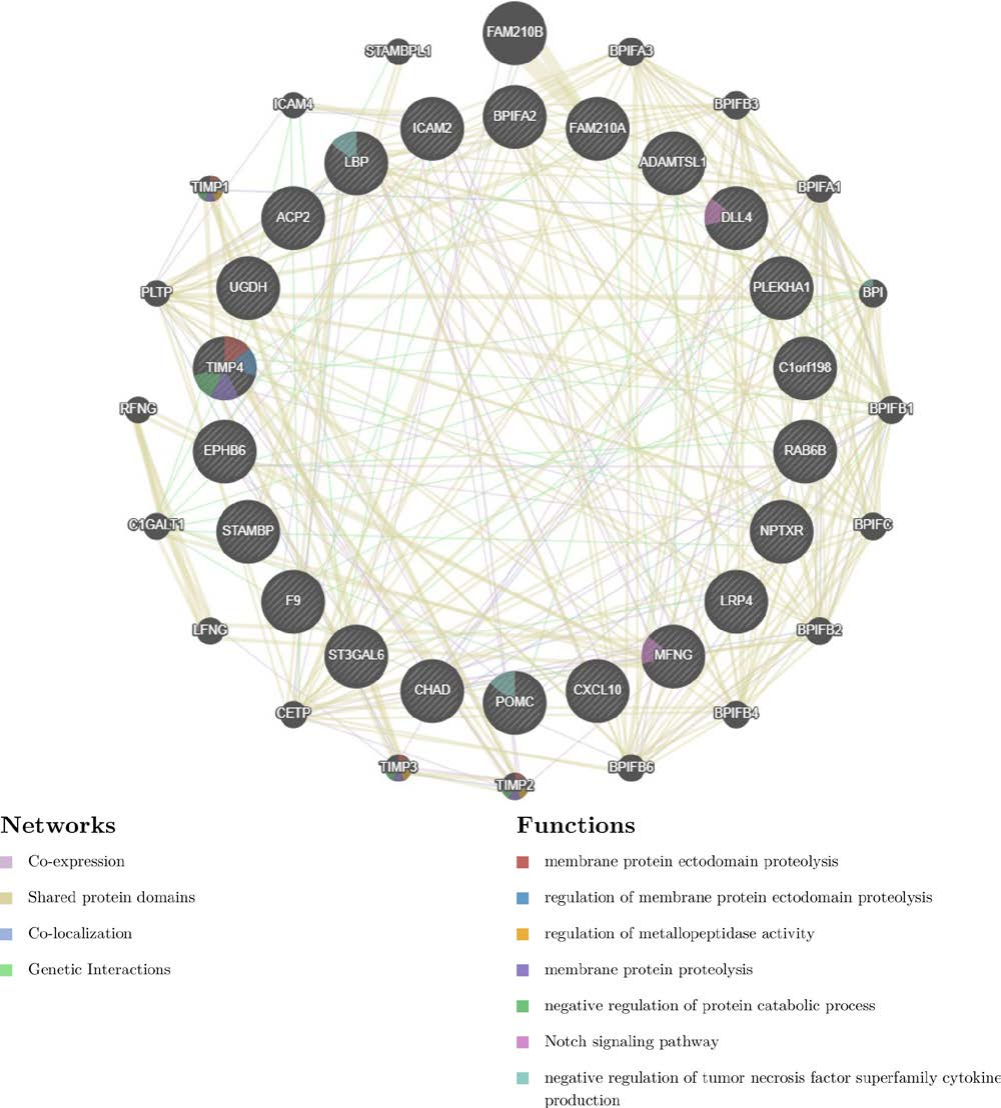
Network map of 22 strongly related plasma protein interaction genes.

To further investigate the biological functions mediated by the 22 plasma proteins, we obtained the interacting proteins as well as the functional enrichment by using tools such as coexpression, shared protein structural domains, colocalization, and gene interactions in the GeneMANIA database. The results showed that the 22 plasma proteins mainly interacted with 20 genes, including tissue inhibitor of metalloproteinase 1 (TIMP1), TIMP2, TIMP3, phospholipid transfer protein (PLTP), lunatic fringe (LFNG), and so forth; they were primarily involved in Notch signaling pathway, protein hydrolysis of extracellular domains of membrane proteins, and the regulation of metallopeptidase activity, and so forth. The GeneMANIA interaction network diagram is shown in Fig. 9.

### 3.6. Selection of key variables for prediction

**Figure 10.**
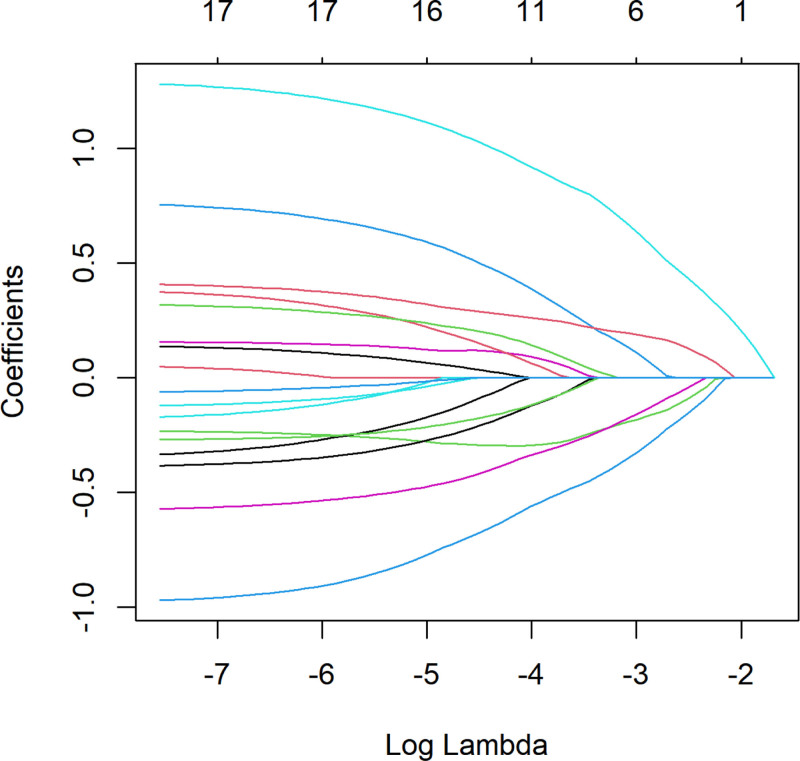
The LASSO coefficient path of predictive factors. LASSO = least absolute shrinkage and selection operator.

**Figure 11.**
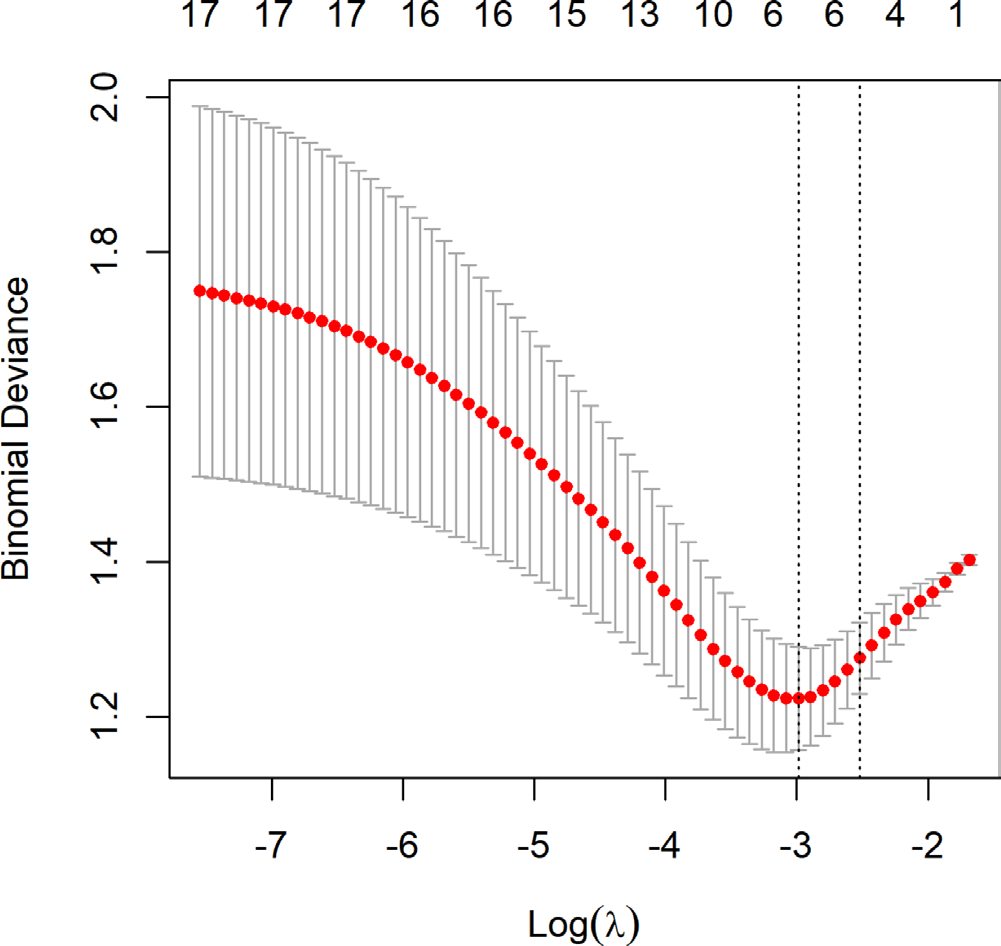
Cross-validation curve (λ.min = 0.050389996, λ.1se = 0.080235124).

All risk factors were subjected to LASSO regression for dimensionality reduction, extracting the most important predictive factors and eliminating collinearity to avoid overfitting. The optimal parameter (lambda) in the final LASSO model was selected through cross-validation with lambda.1se chosen as the optimal value (*λ* = 0.080235124). The LASSO regression results showed that the 6 key predictive variables were UGDH, manic fringe homolog (MFNG), CHAD, RAB6B, member RAS oncogene family (RAB6B), lipopolysaccharide-binding protein (LBP), and recombinant pleckstrin homology domain containing family A (PLEKHA1). Figures 10 and 11, respectively, display the LASSO coefficient path plot and cross-validation curve of the predictors.

## 4. Discussion

OP has become one of the most prevalent diseases in the world, with serious impacts on people's daily lives and huge socioeconomic implications.^[27]^ Despite its high prevalence, it is often overlooked in its early stages, and the later stages of the disease usually lead to vertebral fractures, progressive spinal deformities, and neurological complications.^[28]^ Therefore, the search for OP-specific diagnostic markers is crucial for exploring the pathogenesis of OP as well as for drug development and treatment. Plasma, one of the most abundant biofluids in the human body, contains 1000s of proteins that reflect the body's state of health, disease processes, and therapeutic responses. Therefore, plasma proteomics has great potential for the diagnosis, treatment, and prognostic assessment of diseases. Molecular research and bioinformatics technologies have developed rapidly in recent decades, and through molecular enrichment to analyze functions, BPs, and cellular components, molecular biology can provide clues for comprehensive and further studies of how genetic variation and coexpression affect protein function and disease progression.^[29]^

In the present study, we screened 4907 plasma proteins for genetic causality between plasma proteins and BMD using MR and combined with bioinformatics tools and screened plasma proteins that were strongly correlated with BMD by the *F* test value, with the thresholds set at *P* < .05, an FDR value of <0.2, and a consistent OR value for all 5 statistical methods, and a total of 22 plasma protein genes were obtained. Among them, STAM-binding protein, LRP4, PLEKHA1, EPHB6, RAB6B, DLL4, UGDH, proopiomelanocortin, and ADAMTS-like 1 were risk factors for OP, whereas ST3 β-galactoside α2,3-sialyltransferase 6, LBP, NPTXR, acid phosphatase 2, TIMP4, BPI fold containing family A member 2, CXC motif chemokine ligand 10, C1orf198, intercellular adhesion molecule 2, MFNG, FAM210A, F9, and CHAD were protective factors for OP.

Twenty-two plasma proteins were analyzed by GO enrichment, and their BPs were mainly enriched in the Notch signaling pathway, trabecular morphogenesis, and so forth. KEGG was primarily enriched on the Notch signaling and TLR signaling pathways. Several reports have demonstrated that the Notch signaling pathway,^[30]^ bone trabecular morphogenesis,^[31]^ and TLR signaling pathway are all closely related to the development of OP.^[32]^ It is well known that the structure and function of bone trabeculae depend on the dynamic balance between osteoblast formation and osteoclast resorption. Osteoblasts dominate the synthesis and mineralization of bone matrix, osteoclasts are responsible for the resorption and renewal of bone tissue, and osteocytes regulate this process by sensing mechanical signals. All 3 work in synergy to maintain bone health, and their imbalance will lead to OP, fractures, and other diseases. The Notch signaling pathway is assembled and triggered by a complex mechanism. First, the Notch receptor protein is synthesized in the endoplasmic reticulum as a type I transmembrane protein and then translocated to the cell surface to form a heterodimer; subsequently, the binding of ligand from the signal-sending cell to the extracellular structural domain of the Notch receptor in the signal-receiving cell, or the activation of the nonligand-dependent Notch receptor, results in the dissociation of the receptor extracellular subunit and its transmembrane subunit, which leads to release of the activated intracellular structural domain of Notch receptors; finally, the intracellular structural domain of Notch receptors enters the nucleus and complexes with other proteins to form a transcription complex, thereby regulating gene transcription. Finally, the Notch receptor intracellular domain enters the nucleus and complexes with other proteins to form a transcription complex that regulates gene transcription.^[33]^ Multiple studies have shown that the Notch pathway has multiple regulatory functions during skeletal development and bone remodeling.^[34]^ Skeletal stem and progenitor cells (SSPC) perform skeletal maintenance and repair. With age, they produce fewer osteoblasts and more adipocytes, leading to loss of skeletal integrity. In contrast, Notch regulates SSPC cell fate decisions, and modulation of Notch signaling improves the skeletal aging phenotype and increases bone mass even beyond that of young mice.^[35]^ TLRs can specifically recognize ligands, which subsequently induce the expression of a variety of genes and activate intracellular innate immunity.^[36]^ TLRs, as typical representatives of intrinsic immune pattern recognition receptors, are widely involved in the recognition of pathogen-related molecular patterns and the perception of endogenous danger signal-related molecular patterns, which in turn initiate intrinsic immune responses.^[37]^ Bone homeostasis is maintained due to the interplay of osteoblast-involved osteogenesis and osteoclast-involved bone resorption, and previous reports have confirmed that TLRs are abundantly expressed on the surface of osteoblasts, osteoclasts and osteoclasts and are involved in mediating bone loss.^[38,39]^ It has been found that a reduction in TLR9, a member of the TLR family, significantly increases BMD, bone volume/tissue volume, and trabecular number, thereby promoting fracture recovery.^[40]^

GeneMANIA showed that TIMP1, TIMP2, TIMP3, PLTP, and LFNG interacted strongly with the plasma proteins obtained in this study. TIMP1, TIMP2, and TIMP3 have been reported to be involved in bone metabolism, and the inhibition of TIMP1 expression accelerated the apoptosis of osteoblasts and promoted OP.^[41–44]^ It is well known that OP-induced bone loss must be accompanied by an increase in bone marrow adipose tissue, and inhibition of the bone marrow adipose tissue replacement process is the most promising therapeutic modality for OP, and elevated PTLP expression promotes fatty tissue production,^[45]^ Despite the lack of studies directly correlating PTLP with OP, I cannot ignore its great potential. As one of the molecules in the Notch signaling pathway, it has been demonstrated that messenger RNA levels of Notch pathway molecules, such as LFNG, Notch1, and Nfatc1 were elevated in ovariectomized mice and that Icariin significantly reduced the expression of these genes and inhibited the Notch signaling pathway, and promoted osteogenic differentiation in vitro as well as attenuated the onset of OP in mice.^[46]^ Based on the predicted interactions, we hypothesized that the 22 plasma proteins TIMP4, DLL4, intercellular adhesion molecule 2, UGDH, MFNG, acid phosphatase 2, and BPI fold containing family A member 2 may mediate OP by regulating the genes TIMP1, TIMP2, TIMP3, PLTP, and LFNG.

Six key plasma proteins were further selected using LASSO regression.PLEKHA1 was found to be closely related to the phosphatidylinositol-3-kinase and protein kinase B signaling pathway, which plays an important role in OP.^[47,48]^ Yuan et al^[49]^confirmed that PLEKHA1 is one of the most important network hub genes for OP identified in their experimental study and that it highly interacts with JAG1, an OP-related gene that has been reported to be involved in normal trabecular bone formation. However, no experimental studies have confirmed the direct targeting of PLEKHA1 for OP. However, Hu et al^[49]^integrated multi-omics data and similarly found that PLEKHA1 is a key pleiotropic gene for OP development.

CHAD is a cartilage matrix protein that mediates the adhesion of detached chondrocytes. Its protein core consists of 11 leucine-rich repeats flanked by cysteine-rich structural domains.CHAD interacts importantly with collagen as well as with cell surface heparan sulfate proteoglycans and α2β1 integrins.^[50]^ Capulli et al^[51]^ observed lower CHAD messenger RNA and protein in bone sample biopsies from osteoporotic women and ovariectomized mice between the ages of 50 and 65 years, which has potential translational implications for the treatment of OP by decreasing osteoclasts and bone resorption through cyclic CHAD without affecting osteoblast parameters and bone formation. In this study, GO enrichment analysis suggests that CHAD is associated with trabecular morphogenesis, potentially influencing the progression of OP by regulating trabecular morphogenesis.

LBP is a key inflammatory marker whose main function is to bind lipopolysaccharide and promote its clearance.^[52,53]^ Research findings indicate that LBP can enhance the binding of lipopolysaccharide to CD14 and TLR4 receptors, regulate cytokines, including tumor necrosis factor alpha, and induce osteoclast formation and function.^[54,55]^ In this study, LBP was considered a protective factor for OP, showing a negative correlation with the development of OP. In a recent study by Cleminson et al^[56]^, their team conducted a follow-up study on BMD data of 1149 men. Although they found a negative correlation between LBP and BMD in the mid-forearm, no significant associations were observed in the spine, whole body, or other sites. Further research is needed to explore the role of LBP in skeletal health.

The human MFNG gene is located on chromosome 22, band q13.1, encoding a protein with a relative molecular mass of 36,000. Currently, there are limited studies on its relationship with bone metabolism. The protein encoded by MFNG modifies EGF, which can bind to DLL1 to activate Notch1 while inhibiting the activation of Notch1 by Jag1.^[57–59]^ Previous studies have found that the Notch1 signaling pathway plays a crucial role in the pathophysiological process of OP by regulating the dynamic balance between osteoblasts and osteoclasts. RAB6B is an isomer protein of the RAB6 subfamily, primarily located in the Golgi complex, and has been found to be associated with the development of various cancers.^[60]^ Studies have shown that RAB6B may be involved in the regulation of the inflammatory factor tumor necrosis factor alpha, which participates in osteoclast differentiation and affects the balance between bone formation and resorption.^[47,61]^ UGDH is a cytoplasmic hexameric enzyme that converts UDP-glucose to UDP-glucuronic acid, a key reaction in hormone and exogenous metabolism as well as in the production of extracellular matrix precursors, which plays an important role in a wide range of human cancers including lung, breast, esophageal, hepatocellular, prostate, ovarian, colorectal, and melanoma.^[62]^ RAB6B and UGDH have been extensively studied in cancer research, while MFNG is mainly found to be associated with Notch1. No studies have been reported on these 3 genes in OP. In contrast, this study identified EPHB6 and UGDH as risk factors for OP development. Conversely, MFNG is considered a protective factor requiring further research and clinical validation.

Although this study's plasma protein exposure data were derived from European populations, the genetic mechanisms and causal relationships provide important references for OP research in Asian populations. Due to the racial specificity of genetic variants, the effectiveness of IVs, and limitations of cross-population causal inference, the results of this study are partially applicable to Asian populations. However, the findings of this study also have multifaceted promoting effects on OP research in Asian populations. This study can serve as one of the starting points for Asian research, guiding research directions, providing methodological references for the relationship between plasma proteins and OP in Asian populations, revealing racial differences, promoting research on Asian-specific mechanisms, facilitating multi-omics and interdisciplinary integration, advancing clinical translation and precision medicine, and promoting drug development.

## 5. Conclusion

In this study, we used an integrative approach to reveal a genetic susceptibility association between plasma proteins and OP risk. We obtained 22 plasma proteins that can be used as diagnostic and therapeutic targets for OP, which have great potential for OP diagnosis and treatment but need to be subsequently and continuously validated in the clinic. Our findings provide new insights into the diagnosis and treatment of OP and highlight potential targets that should be prioritized in the future in terms of early diagnosis and therapeutic strategies for OP.

This study has some limitations, as all GWAS data were derived from European populations. It is worth exploring whether similar genetic variants exist in other populations, and large databases should be utilized in the future to obtain cross-ethnic GWAS data to improve the generalizability of the findings. Second, the MR analysis in this study set slightly relaxed FDR values in the hope that more disease-related plasma proteins could be screened out, which is more common in other studies, and the *F*-statistic values of all selected SNPs were >10, suggesting that the IV was sufficiently robust. Finally, the level of evidence for MR was higher than that of traditional observational studies but still lower than that of randomized controlled trials, and its results need to be experimentally validated to exclude methodological limitations, confirm biological mechanisms, and provide a basis for clinical translation.

## Author contributions

**Conceptualization:** Cai Chen, Qin Zeng.

**Data curation:** Cai Chen, Qianling Ye, Futai Jin.

**Formal analysis:** Cai Chen, Qin Zeng, Qianling Ye.

**Funding acquisition:** Cai Chen.

**Methodology:** Cai Chen, Qianling Ye.

**Software:** Cai Chen.

**Writing – original draft:** Cai Chen.

**Writing – review & editing:** Cai Chen, Qin Zeng, Qianling Ye.
